# POLR3A‐related disorders: From spastic ataxia to generalised dystonia and long‐term efficacy of deep brain stimulation

**DOI:** 10.1002/acn3.52064

**Published:** 2024-05-03

**Authors:** Wai Yan Yau, Catherine Ashton, Eoin Mulroy, Thomas Foltynie, Patricia Limousin, Jana Vandrovcova, Kunal P. Verma, Rick Stell, Mark Davis, Phillipa Lamont

**Affiliations:** ^1^ Perron Institute for Neurological and Translational Science The University of Western Australia Nedlands Western Australia Australia; ^2^ Department of Neurology Royal Perth Hospital Perth Western Australia Australia; ^3^ Department of Clinical and Movement Neurosciences UCL Queen Square Institute of Neurology London UK; ^4^ Unit of Functional Neurosurgery UCL Queen Square Institute of Neurology, National Hospital for Neurology and Neurosurgery London UK; ^5^ Department of Neuromuscular Diseases UCL Queen Square Institute of Neurology London UK; ^6^ Baker Heart and Diabetes Research Institute Melbourne Victoria Australia; ^7^ Baker Department of Cardio‐Metabolic Health University of Melbourne Melbourne Victoria Australia; ^8^ Monash Heart Melbourne Victoria Australia; ^9^ Department of Diagnostic Genomics PathWest Laboratory Medicine, West Australian Department of Health Nedlands Western Australia Australia

## Abstract

While biallelic *POLR3A* loss‐of‐function variants are traditionally linked to hypomyelinating leukodystrophy, patients with a specific splice variant c.1909+22G>A manifest as adolescent‐onset spastic ataxia without overt leukodystrophy. In this study, we reported eight new cases, *POLR3A*‐related disorder with c.1909+22 variant. One of these patients showed expanded phenotypic spectrum of generalised dystonia and her sister remained asymptomatic except for hypodontia. Two patients with dystonic arm tremor responded to deep brain stimulation. In our systemic literature review, we found that *POLR3A*‐related disorder with c.1909+22 variant has attenuated disease severity but frequency of dystonia and upper limb tremor did not differ among genotypes.

## Introduction

*POLR3A*‐related disorders encompass neonatal progeroid syndrome, childhood‐onset hypomyelinating leukodystrophies to adult‐onset spastic ataxia.^1^, ^2^, ^3^, ^4^ Specific clinical features such as hypodontia, hypogonadotropic hypogonadism and myopia are variably present depending on the underlying genetic variants. In 2017, a study by Minnerop et al. reported a specific phenotype of adolescent‐onset progressive spastic ataxia and tremor due to a *POLR3A* splice variant c.1909+22G>A in trans with another loss‐of‐function variant.^3^ The neuroimaging of these patients demonstrated hyperintensity along superior cerebellar peduncles (SCP) in T2 and FLAIR sequences on MRI brain without overt hypomyelination. This specific variant also causes Wiedemann‐Rautenstrauch syndrome.^4^ Individuals with *POLR3A* c.1909+22 variant represent one of the most common causes of autosomal recessive ataxia and over half of reported cases demonstrated postural/kinetic tremor of upper limbs.^5^ However, there are no published data on clinical response of tremor to deep brain stimulation (DBS). In this study, we present the expanded phenotypic spectrum in eight new patients harbouring *POLR3A* c.1909+22 variant and in two of these patients we reported their DBS outcomes. Through a systemic literature review, we compared phenotypically individuals carrying this specific variant versus those without it.

## Methods

We identified cases of POLR3A‐associated disorders with c.1909+22G>A variant from PathWest Neurogenetics laboratory Western Australia and the Neurogenetics unit at the National Hospital for Neurology and Neurosurgery United Kingdom. Sequencing methods included gene panels and whole‐exome sequencing previously described.^6^, ^7^ Family members enrolled for segregation studies underwent variant‐specific Sanger sequencing. Literature search on PubMed (15 January 2023) for POLR3A‐related disorders included terms “POLR3A” or “RNA polymerase III” or “4H leukodystrophy” or “Wiedemann‐Rautenstrauch Syndrome” or “HLD 7” or “TACH” or “ADDH” or “HCAHC.” We included patients carrying biallelic POLR3A variants with available clinical information. Predefined categories for data extraction were sex, age at onset, age at last assessment, family history, neurological and non‐neurological symptoms/signs, neurophysiological tests, neuroimaging and genotype. Phenotypic features were recorded as missing if not explicitly stated. POLR3A variants were annotated to GRCh38 transcript NM_007055.4. Population frequency was obtained from gnomAD.^8^ In silico tools used to predict the impact of missense mutations on the protein structure and functions were Combined Annotation Dependent Depletion, PolyPhen‐2, Sorting Intolerant From Tolerant. Variants were classified according to American College of Medical Genetics and Genomics guidelines.^9^ Comparisons between two groups were tested for statistical significance using a chi‐squared test for categorical variables and Mann–Whitney *U*‐test for continuous variables. Bonferroni correction was used for P‐values. Analyses were performed using R version 3.6.1. This study was approved by the University of Western Australia Human Research Ethics Committee (RA/4/20/1008).

## Results

We identified eight patients who carry c.1909+22G>A *POLR3A* variant and another pathogenic variant in trans. The mean age of motor onset was 23 years (range: 8–38 years). The distribution of sex was equal. All their ethnicity was Caucasian. The most common presenting complaint was gait imbalance (*n* = 4), followed by upper limb tremor (*n* = 2) and head tremor (*n* = 1). The inheritance patterns were autosomal recessive in two families and sporadic for the remaining patients. The predominant phenotypes were spastic ataxia (*n* = 3), segmental dystonia with ataxia (*n* = 2), spastic paraplegia (*n* = 1), generalised dystonia (*n* = 1), and asymptomatic (*n* = 1). The most common neurological features were cerebellar ataxia (*n* = 5, 63%), spasticity (*n* = 5, 63%), dystonia (*n* = 4, 50%), and upper limb action tremor (*n* = 3, 38%). Two of the patients with upper limb tremor underwent DBS due to their severity. None had parkinsonism, myoclonus, chorea or cognitive/motor developmental delay. In non‐neurological features, three patients had hypodontia but no one had hypo‐gonadotrophic hypogonadism.

**Figure 1:**
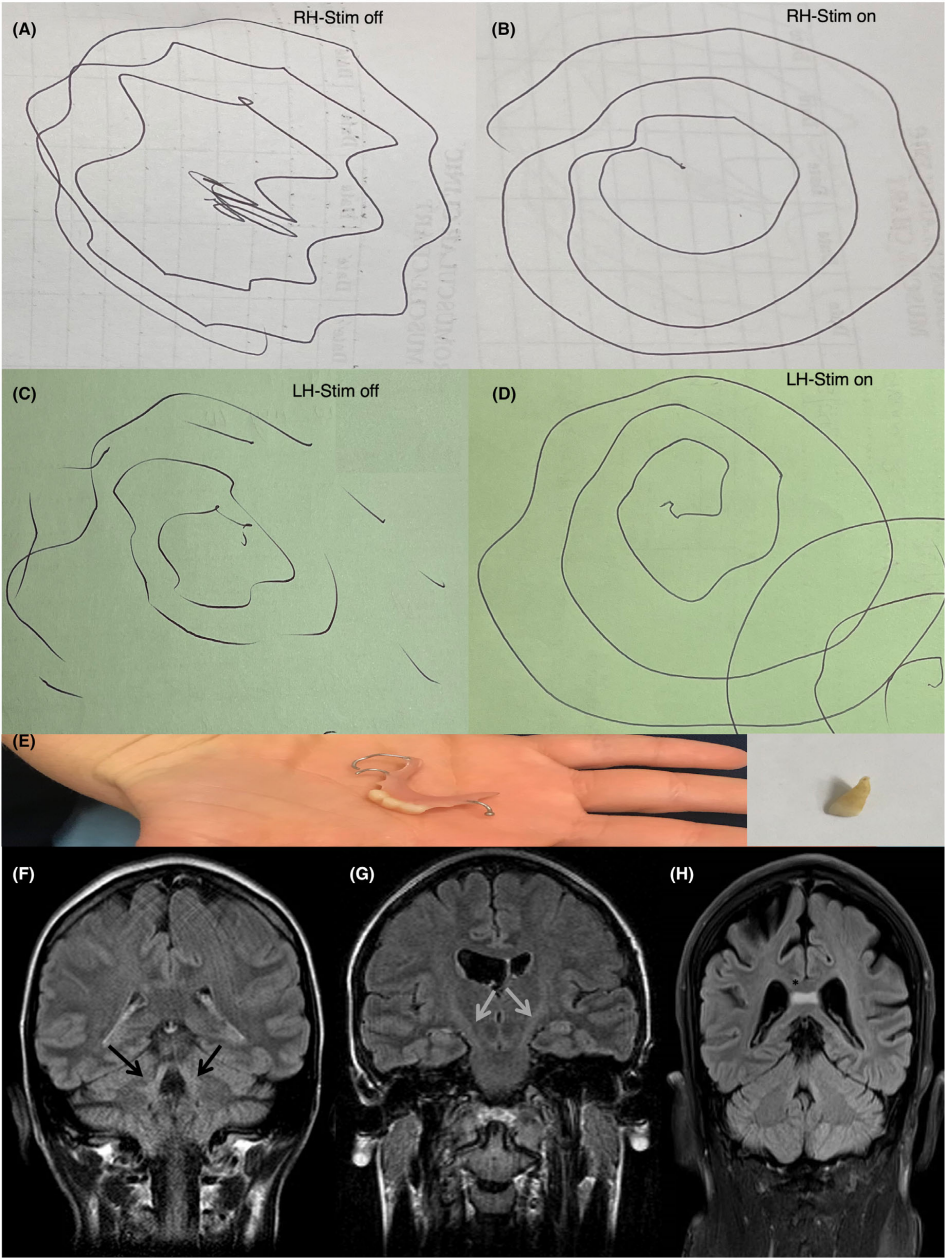
Archimedean spiral of patient D1 with DBS on/off (A–D); denture of patient D2 with insert showing her one of her small teeth (E); MRI head coronal FLAIR of patient F1 showing SCP sign (F), patient D1 showing pyramidal tract hyperintensities (G), and patient C1 showing splenium of corpus callosum hyperintensities (H).

MRI brain were abnormal in half of patients (Table 1, Fig. 1F–H). The scans of patient F1 displayed T2 and FLAIR hyperintensities in pyramidal tracts, midbrain, dorsal pons and SCP. These changes remain stable over 12 years of follow‐up, discordant to her clinical progression. The scans of patient D1 also showed pyramidal tract T2 hyperintensities without SCP involvement while that of patient C:1 showed hyperintensities in splenium of corpus callosum. Patient E1 neuroimaging demonstrated brainstem atrophy alone. Further clinico‐radiologico‐genetic features of these patients are available in Table 1.

**Table 1:** Clinical and genetic summaries of patients carrying POLR3A C.1909+22G>A variant.

	A:1	B:1	C:1	D:1	D:2	E:1	E:2	F:1
*Patient*								
Sex	M	M	M	F	F	F	M	F
Age at onset	14	38	25	8	n/a	25	23	28
Age at evaluation	59	70	40	43A	28	48	47	56
Presenting symptoms	UL tremor	Gait imbalance	Gait imbalance	UL tremor	n/a	Gait imbalance	Gait imbalance	Head tremor
Inheritance	Sporadic	Sporadic	Sporadic	AR	AR	AR	AR	Sporadic
*Clinical features neurological*								
Ataxia‐limb	Y mild	Y severe	N	N	N	N	N	Y mild
Ataxia‐gait	Y mild	Y severe	N	N	N	Y mod	Y mod	Y mod
Cerebellar eye movements	N	Y	N	N	N	Y	Y	Y
Parkinsonism	N	N	N	N	N	N	N	N
UL action tremor	Y severe	N	N	Y severe	N	N	N	Y mild
Dystonia	Y cervical	N	Y cervical	Y generalised	N	N	N	Y cervical
Spasticity	Y mild, LL	Y mod, UL+LL	Y mod, LL	N	N	Y mod, LL	Y mod, LL	N
Plantar response	Upgoing	Upgoing	Upgoing	Downgoing	Downgoing	Upgoing	Upgoing	Downgoing
Peripheral neuropathy	N	N	N	N	N	N	N	N
Dysarthria	Y	Y mod	N	Y mild	Y mild	Y mild	Y mild	Y mild
Dysphagia	N	Y mild	N	N	N	N	N	N
Developmental delay	N	N	N	N	N	N	N	N
*Non‐neurological*								
Dental abnormalities	N	N	N	Y hypodontia	Y hypodontia	N	N	Y hypodontia
Hypo‐gonadotrophic hypogonadism	N	N	N	N	N	N	N	N
Myopia	N	N	N	N	Y	N	N	N
*Investigations*								
NCS	Normal	n/a	n/a	Normal	Normal	n/a	n/a	n/a
MRI	Normal, not available for review	Normal, not available for review	Corpus callosum hyperintensity	Pyramidal tract hyperintensity	Normal	Brainstem atrophy	n/a	Leukodystrophy in pyramidal tract, midbrain, posterior fossa
*Genetics*								
Non +22 variant	NM_007055.4:c.1114G>A	NM_007055.4:c.2181C>A	NM_007055.4:c.3796A>C	NM_007055.4:c.2563delC	NM_007055.4:c.2563delC	NM_007055.4:c.644A>G	NM_007055.4:c.644A>G	NM_007055.4:c. c.2404C>T
CADD score	26.3	40	28.6	n/a	n/a	24.3	24.3	n/a
Sift	0.01	n/a	0	n/a	n/a	0.14	0.14	n/a
Polyphen	0.988	n/a	0.973	n/a	n/a	0.179	0.179	n/a
Predicted consequence	Missense	Stop gained	Missense	Indel	Indel	Missense, splice site	Missense, splice site	Stop gained
Allele frequency in gnomAD	0.0001163	0	0	0.00000795	0.00000795	0.000004	0.000004	0
Variant previously reported in Clinvar	Y	N	N	N	N	N	N	N
Estimated consequence as per ACMG	Likely pathogenic	Pathogenic	Likely pathogenic	Pathogenic	Pathogenic	Likely Pathogenic	Likely Pathogenic	Pathogenic

AR, autosomal recessive; LL, lower limb; MRI, magnetic resonance imaging; N, no; n/a not available; NCS, nerve conduction study; UL, upper limb; Y, yes; cerebellar eye movements: saccadic intrusion, gaze‐evoked nystagmus or hyper−/hypometria.

### Case studies

Patient A1 is a 60‐year‐old white British man with multifocal dystonia and spastic ataxia. His symptoms started at age 14 years with head and neck tremor, which was alcohol responsive. Subsequently he developed upper limb tremor and dysarthria in his twenties. His lower limb spasticity began in his forties along with urinary urgency/incontinence. His family tree is in Fig. S1. Examination revealed torticollis to left with no‐no head tremor, upper limb postural and action tremor worse on the right, dystonic posturing of right wrist. He was unable to copy a spiral nor write. His lower limb tone was increased with extensor plantar responses. His cervical dystonia was helped by botulinum toxin but not trihexyphenidyl. His upper limb tremor did not respond to propranolol. Pallidal DBS (DBS‐GPi) with Medtronic Activa RC at age 47 years achieved partial improvement in speech (40%), drawing spiral, upper limb tremor and functional tasks such as eating and drinking; benefits gradually waned over 14 years. His DBS settings for GPi were 0‐1‐:2 V/60 μs/130 Hz and 8‐9‐:2 V/60 μs/130 Hz.

Patient D1 is a 45‐year‐old white Australian woman with generalised dystonia and prominent upper limb action tremor. Her arm tremor began at age 8 years old and progressed over the years interfering with her ability to perform activities of daily living. Subsequently she developed abnormal posturing in trunk and lower limbs in her thirties. She reported microdontia in childhood. Her family tree is in Fig. S2. Examination revealed dystonic posturing of upper limbs and left greater than right postural and intention tremor. She had a narrow‐based gait and right truncal tilt but tone, power, and cerebellar function were normal (Videos [Link], [Link], [Link]). Oral trihexyphenidyl, baclofen, clonazepam, and propranolol were not effective. Initial DBS‐GPi with Medtronic Activa RC at age 25 years did not suppress her tremor (Fig. 1A–D). Repositioning of the leads to ventralis intermediate nucleus of thalamus (Vim) completely suppressed the tremor with gradual waning but sustained benefits over 20 years. Unified dystonia rating scale was reduced from 16 to 10. Her DBS settings for Vim were C+1‐: 2 mA/90 μs/130 Hz and C+9‐: 2.3 mA/90 μs/130 Hz. Patient D2 is her younger sister who was diagnosed with Down's syndrome since birth. She only manifested with microdontia but no additional clinical features (Fig. 1E) (Video S4).

### Review of published studies

We included 208 patients with biallelic *POLR3A* variants from 42 cases series/reports for analysis (Fig. S3): 76 individuals with at least one c.1909+22 variant, 132 individuals without the c.1909+22 variant (Table S1). Sexes were evenly split. The median age of onset was 13 years (range: 0–51 years). Sixty‐one out of 207 patients have family history consistent with autosomal recessive inheritance. Most common neurological features in descending orders were spasticity (125 out of 133, 94%), cerebellar ataxia (156 out of 187, 83%), epilepsy (50 out of 89, 56%), dystonia (56 out of 139, 40%), upper limb action tremor (42 out of 108, 39%), and parkinsonism (10 out of 46, 22%). Optic atrophy and chorea were rarely reported. Dental abnormalities (microdontia/hypodontia) (116 out of 177, 66%), myopia (67 out of 126, 53%), and craniofacial abnormalities (34 out of 64, 53%) were the most prevalent non‐neurological features. Hypogonadotrophic hypogonadism was only present in 18 out of 105 (17%). Neuroimaging abnormalities were common, these included hypomyelination (90 out of 113, 80%), cerebellar atrophy (85 out of 144, 59%), thin corpus callosum (76 out of 132, 58%), cervical cord atrophy (37 out of 72, 51%), SCP hyperintensity (59 out of 136, 43%), and striatal abnormalities (25 out of 208, 12%).

**Figure 2:**
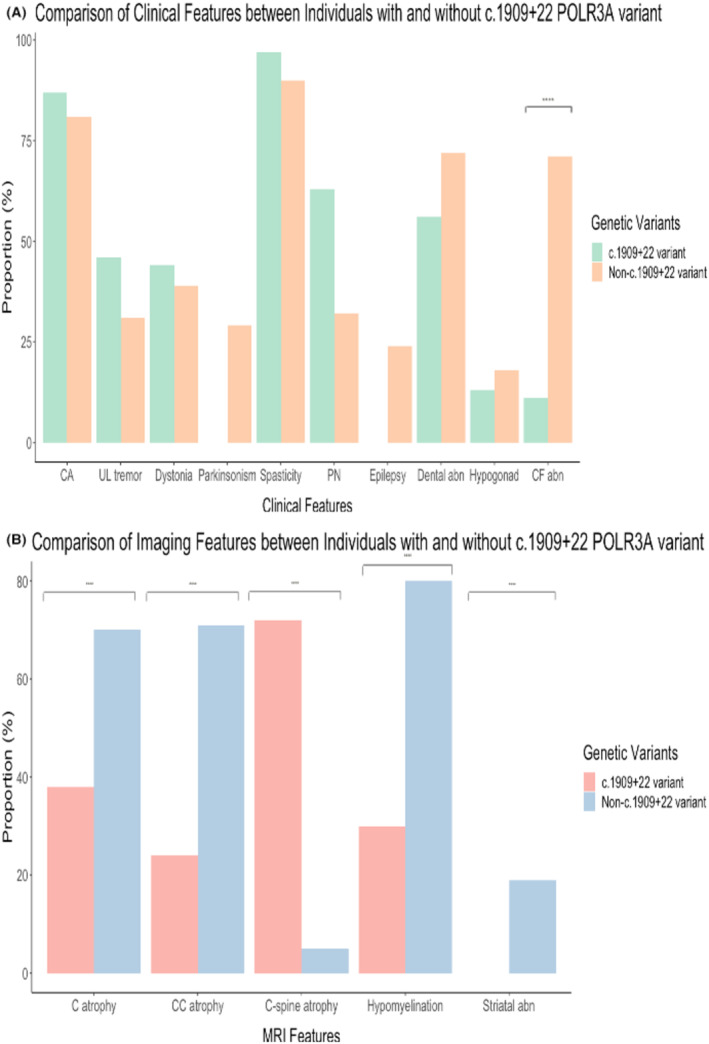
Comparison of (A) clinical features and (B) radiological features between individuals with and without the splice variant. **** denotes *p* < 0.001.

Compared to patients carrying one copy of c.1909+22 POLR3A variant, patients in whom both variants were non‐c.1909+22 variants were associated with an earlier age of symptom onset, a positive family history, craniofacial abnormalities, motor and cognitive developmental delay, dysarthria, and dysphagia (Fig. 2). With neuroimaging, SCP T2/FLAIR hyperintensities and atrophic cervical cord were more prevalent in patients with one copy of c.1909+22 variant. In contrast, thin corpus callosum, hypomyelination, and striatal abnormalities were associated more often with non‐c.1909+22 variants.

## Discussion

We provided detailed phenotypic and genotypic characterisation of eight patients harbouring *POLR3A* c.1909+22G>A variant. Our results confirmed previous studies that the predominant clinical syndrome was spastic ataxia with high prevalence of upper limb action tremor and dystonia and few extra‐neurological features.^3^, ^10^ However, patients in our series did not have peripheral nerve involvement. In those whose neuroimaging were available, MRI head demonstrated typical radiological features of SCP and pyramidal tract hyperintensities. Our study reported an expanded phenotypic spectrum of patients with this specific *POLR3A* variant: a proband (D1) demonstrated childhood‐onset pure generalised dystonia with severe upper limb action tremor while her sister (D2) was asymptomatic except for hypodontia. Together with another patient with spastic ataxia and upper limb tremor(A1), we showed that DBS‐Vim and GPi could be effective treatment for tremor/ dystonia. To date, only one patient with *POLR3A*‐related leukodystrophy and parkinsonism was reported to have response to pallidal nucleus DBS.^11^

While dystonia and tremor are common symptoms in patients with *POLR3A*‐related hypomyelinating leukodystrophy, they are often under‐recognised.^12^, ^13^ The attenuated disease severity associated with c.1909+22 variant enables DBS to become a potential symptomatic treatment option for severe tremor or dystonia. The findings in our systemic review also aligned with two previous studies^5^, ^14^: *POLR3A* c.1909+22 variant is associated with a distinct neurological phenotype with wide clinical heterogeneity and intra‐familial variability. This is especially important in the context of emerging clinical trials for splice‐modulating therapies. Limitations of this study are mainly attributable to its retrospective nature and incomplete clinicoradiological feature reporting in published case series, which may lead to underestimation of these features.

In conclusion, a specific combination of biallelic *POLR3A* mutations with the c.1909+22G>A variant typically causes spastic ataxia but it can also manifest as generalised dystonia with prominent upper limb tremor that is DBS responsive.

## Author Contributions

WYY, CA, MD, and PL contributed to conception and design of the study; WYY, CA, EM, TF, PL, JV, KV, RS, MV, and PL contributed to the acquisition and analysis of data; WYY and CA contributed to drafting the text and preparing the figures.

## Conflict of Interest

The authors declare no competing interests.

## Supporting information


Supplementary figure 1.

Supplementary table 1.

Supplementary video 1.

Supplementary video 2.

Supplementary video 3.

Supplementary video 4.

## Data Availability

The supporting findings of this study are available from the corresponding author upon reasonable request.
