# Supplementary material for: POLR3A‐related disorders: From spastic ataxia to generalised dystonia and long‐term efficacy of deep brain stimulation
Source: Ann Clin Transl Neurol. 2024 May 3;11(6):1636–42. doi: 10.1002/acn3.52064 (PMC11187961; doi:10.1002/acn3.52064)
Supplement: Supplementary file 1 — Supplementary figure 1. [file ACN3-11-1636-s003.pdf]

Supplementary figure 1. Family tree of patient A1.

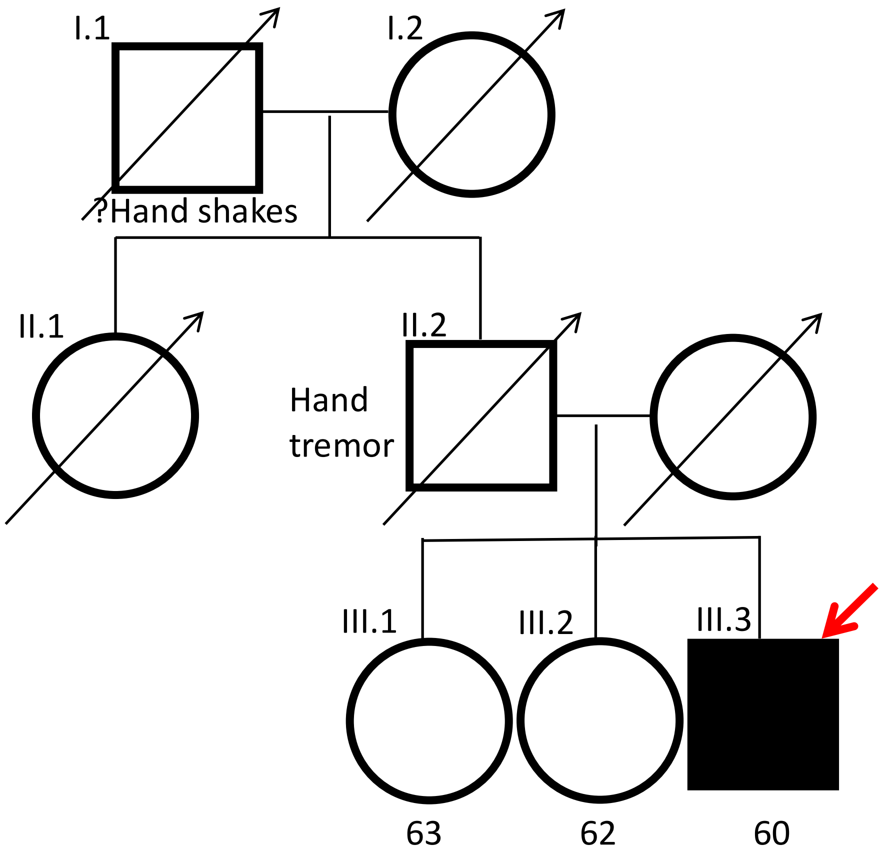

Supplementary figure 2. Family tree of patients D1 and D2.

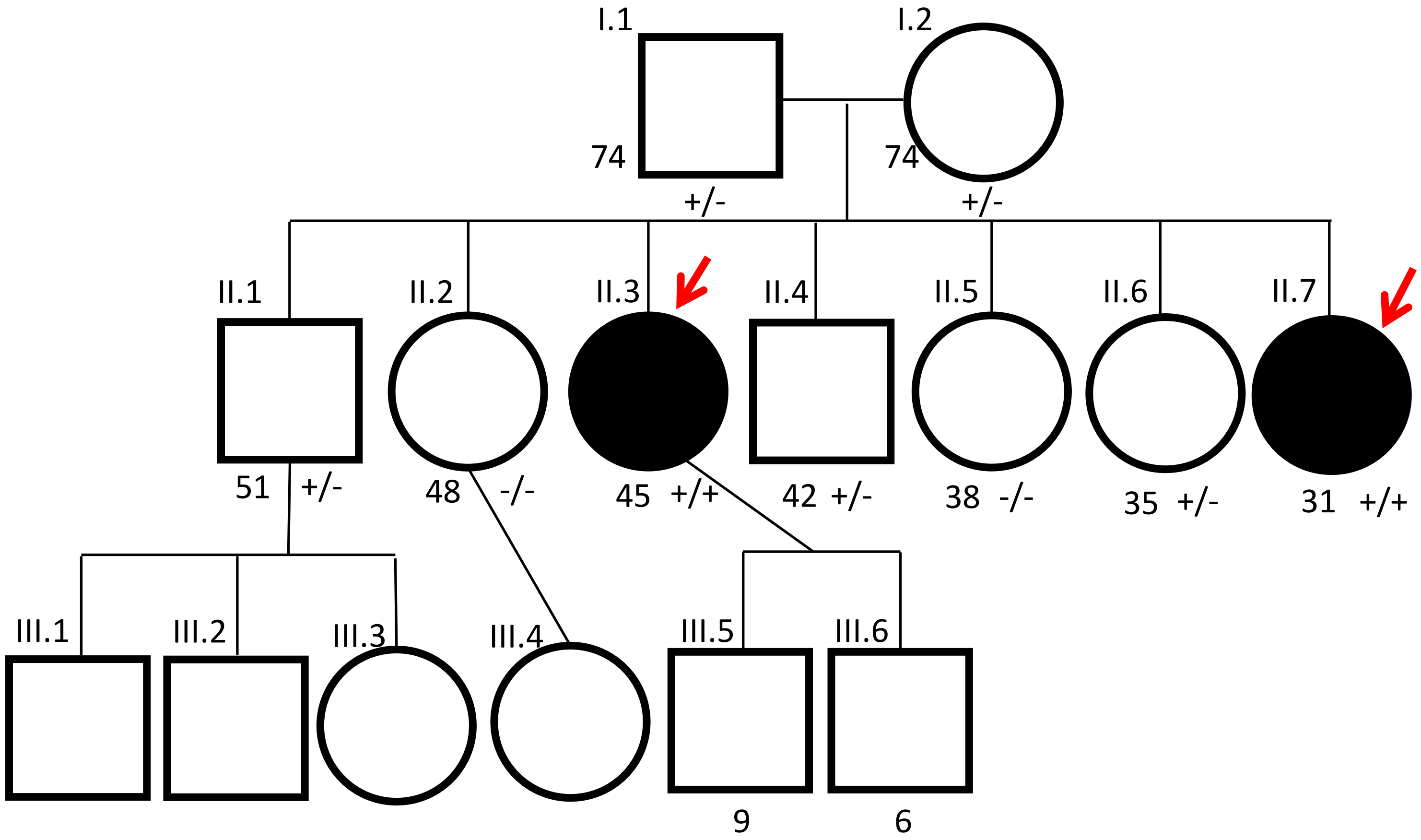

Supplementary figure 3. References for articles included in the systemic review.

1. Wambach JA, Wegner DJ, Patni N, et al. Bi-allelic POLR3A Loss-of-Function Variants Cause Autosomal-Recessive Wiedemann-Rautenstrauch Syndrome. Am J Hum Genet. 2018 Dec 6;103(6):968-75.
2. Wolf NI, Vanderver A, van Spaendonk RM, et al. Clinical spectrum of 4H leukodystrophy caused by POLR3A and POLR3B mutations. Neurology. 2014 Nov 18;83(21):1898-905.
3. La Piana R, Cayami FK, Tran LT, et al. Diffuse hypomyelination is not obligate for POLR3-related disorders. Neurology. 2016 Apr 26;86(17):1622-6.
4. Minnerop M, Kurzwelly D, Wagner H, et al. Hypomorphic mutations in POLR3A are a frequent cause of sporadic and recessive spastic ataxia. Brain. 2017 Jun 1;140(6):1561-78.
5. Di Donato I, Gallo A, Ricca I, et al. POLR3A variants in hereditary spastic paraparesis and ataxia: clinical, genetic, and neuroradiological findings in a cohort of Italian patients. Neurol Sci. 2022 Feb;43(2):1071-7.
6. Infante J, Serrano-Cardenas KM, Corral-Juan M, et al. POLR3A-related spastic ataxia: new mutations and a look into the phenotype. J Neurol. 2020 Feb;267(2):324-30.
7. Rydning SL, Koht J, Sheng Y, et al. Biallelic POLR3A variants confirmed as a frequent cause of hereditary ataxia and spastic paraparesis. Brain. 2019 Apr 1;142(4):e12.
8. Ji H, Li D, Wu Y, et al. Hypomyelinating disorders in China: The clinical and genetic heterogeneity in 119 patients. PLoS One. 2018;13(2):e0188869.
9. Hiraide T, Kubota K, Kono Y, et al. POLR3A variants in striatal involvement without diffuse hypomyelination. Brain Dev. 2020 Apr;42(4):363-8.
10. Azmanov DN, Siira SJ, Chamova T, et al. Transcriptome-wide effects of a POLR3A gene mutation in patients with an unusual phenotype of striatal involvement. Hum Mol Genet. 2016 Oct 1;25(19):4302-14.
11. Kyle K, Mason X, Bordelon Y, Pouratian N, Bronstein J. Adult onset POLR3A leukodystrophy presenting with parkinsonism treated with pallidal deep brain stimulation. Parkinsonism Relat Disord. 2021 Apr;85:23-5.
12. de Assis Pereira Matos PCA, Gama MTD, Bezerra MLE, da Rocha AJ, Barsottini OGP, Pedroso JL. POLR3A-Related Disorder Presenting with Late-Onset Dystonia and Spastic Paraplegia. Mov Disord Clin Pract. 2020 May;7(4):467-9.
13. Harting I, Al-Saady M, Krageloh-Mann I, et al. POLR3A variants with striatal involvement and extrapyramidal movement disorder. Neurogenetics. 2020 Apr;21(2):121-33.

14. Al Yazidi G, Tran LT, Guerrero K, et al. Dystonia in RNA Polymerase III-Related Leukodystrophy. Mov Disord Clin Pract. 2019 Feb;6(2):155-9.
15. Ruggiero L, Iovino A, Dubbioso R, et al. Multimodal evaluation of an Italian family with a hereditary spastic paraplegia and POLR3A mutations. Ann Clin Transl Neurol. 2020 Nov;7(11):2326-31.
16. Lynch DS, Rodrigues Brandao de Paiva A, Zhang WJ, et al. Clinical and genetic characterization of leukoencephalopathies in adults. Brain. 2017 May 1;140(5):1204-11.
17. Lessel D, Rading K, Campbell SE, et al. A novel homozygous synonymous variant further expands the phenotypic spectrum of POLR3A-related pathologies. Am J Med Genet A. 2022 Jan;188(1):216-23.
18. Zea Vera A, Bruce A, Larsh TR, et al. Spectrum of Pediatric to Early Adulthood POLR3A-Associated Movement Disorders. Mov Disord Clin Pract. 2023 Feb;10(2):316-22.
19. Yang YM, Zhao ZM, Jia YL, Jia YJ, Han N, Wang JH. A 42-year-old woman with 4H leukodystrophy caused by a homozygous mutation in POLR3A gene. Chin Med J (Engl). 2019 Aug 5;132(15):1879-80.
20. Yan H, Ji H, Kubisiak T, et al. Genetic analysis of 20 patients with hypomyelinating leukodystrophy by trio-based whole-exome sequencing. J Hum Genet. 2021 Aug;66(8):761-8.
21. Wu S, Bai Z, Dong X, et al. Novel mutations of the POLR3A gene caused POLR3-related leukodystrophy in a Chinese family: a case report. BMC Pediatr. 2019 Aug 22;19(1):289.
22. Lessel D, Ozel AB, Campbell SE, et al. Analyses of LMNA-negative juvenile progeroid cases confirms biallelic POLR3A mutations in Wiedemann-Rautenstrauch-like syndrome and expands the phenotypic spectrum of PYCR1 mutations. Hum Genet. 2018 Dec;137(11-12):921-39.
23. Tewari VV, Mehta R, Sreedhar CM, et al. A novel homozygous mutation in POLR3A gene causing 4H syndrome: a case report. BMC Pediatr. 2018 Apr 4;18(1):126.
24. Campopiano R, Ferese R, Zampatti S, et al. A novel POLR3A genotype leads to leukodystrophy type-7 in two siblings with unusually late age of onset. BMC Neurol. 2020 Jun 29;20(1):258.
25. Yoon Han J, Gon Cho Y, Park J, Jang W. A novel variant of the POLR3A gene in a patient with hypomyelinating POLR3-related leukodystrophy. Clin Chim Acta. 2022 Aug 1;533:15-21.
26. Peng Q, Zhang Y, Xian B, et al. A synonymous variant contributes to a rare Wiedemann-Rautenstrauch syndrome complicated with mild anemia via affecting pre-mRNA splicing. Front Mol Neurosci. 2022;15:1026530.
27. Uygun O, Gunduz T, Eraksoy M, Kurtuncu M. Adult-onset 4H leukodystrophy: a case presentation and review of the literature. Acta Neurol Belg. 2020 Dec;120(6):1461-2.

28. Moon B, Kim M, Kim HJ, et al. Biallelic POLR3A variants cause Wiedemann-Rautenstrauch syndrome with atypical brain involvement. Clin Exp Pediatr. 2023 Mar;66(3):142-4.
29. Morales-Rosado JA, Macke EL, Cousin MA, Oliver GR, Dhamija R, Klee EW. Interpretation challenges of novel dual-class missense and splice-impacting variant in POLR3A-related late-onset hereditary spastic ataxia. Mol Genet Genomic Med. 2020 Sep;8(9):e1341.
30. Zanette V, Reyes A, Johnson M, et al. Neurodevelopmental regression, severe generalized dystonia, and metabolic acidosis caused by POLR3A mutations. Neurol Genet. 2020 Dec;6(6):e521.
31. Baviera-Munoz R, Carretero-Vilarroig L, Vazquez-Costa JF, et al. Diagnostic Efficacy of Genetic Studies in a Series of Hereditary Cerebellar Ataxias in Eastern Spain. Neurol Genet. 2022 Dec;8(6):e200038.
32. Musumeci A, Cali F, Scuderi C, et al. Identification of a Novel Missense Mutation of POLR3A Gene in a Cohort of Sicilian Patients with Leukodystrophy. Biomedicines. 2022 Sep 14;10(9).
33. Saitsu H, Osaka H, Sasaki M, et al. Mutations in POLR3A and POLR3B encoding RNA Polymerase III subunits cause an autosomal-recessive hypomyelinating leukoencephalopathy. Am J Hum Genet. 2011 Nov 11;89(5):644-51.
34. Temel SG, Ergoren MC, Manara E, et al. Unique combination and in silico modeling of biallelic POLR3A variants as a cause of Wiedemann-Rautenstrauch syndrome. Eur J Hum Genet. 2020 Dec;28(12):1675-80.
35. Nikkhah A, Rezakhani S. Developmental regression and movement disorder as a phenotypic variant of POLR3A Mutation-Case report. Clin Case Rep. 2022 Nov;10(11):e6556.
36. Terao Y, Saitsu H, Segawa M, et al. Diffuse central hypomyelination presenting as 4H syndrome caused by compound heterozygous mutations in POLR3A encoding the catalytic subunit of polymerase III. J Neurol Sci. 2012 Sep 15;320(1-2):102-5.
37. Paolacci S, Li Y, Agolini E, et al. Specific combinations of biallelic POLR3A variants cause Wiedemann-Rautenstrauch syndrome. J Med Genet. 2018 Dec;55(12):837-46.
38. Di Bella D, Magri S, Benzoni C, et al. Hypomyelinating leukodystrophies in adults: Clinical and genetic features. Eur J Neurol. 2021 Mar;28(3):934-44.
39. Potic A, Brais B, Choquet K, Schiffmann R, Bernard G. 4H syndrome with late-onset growth hormone deficiency caused by POLR3A mutations. Arch Neurol. 2012 Jul;69(7):920-3.
40. Fellner A, Lossos A, Kogan E, et al. Two intronic cis-acting variants in both alleles of the POLR3A gene cause progressive spastic ataxia with hypodontia. Clin Genet. 2021 May;99(5):713-8.
41. Shimojima K, Shimada S, Tamasaki A, et al. Novel compound heterozygous mutations of POLR3A revealed by whole-exome sequencing in a patient with hypomyelination. Brain Dev. 2014 Apr;36(4):315-21.
42. Majethia P, Girisha KM. Wiedemann-Rautenstrauch syndrome in an Indian patient with biallelic pathogenic variants in POLR3A. Am J Med Genet A. 2021 May;185(5):1602-5.

43. Perrier S, Gauquelin L, Fallet-Bianco C, et al. Expanding the phenotypic and molecular spectrum of RNA polymerase III-related leukodystrophy. Neurol Genet. 2020 Jun;6(3):e425.
